# Derivation and Validation of a 10-Year Risk Score for Symptomatic Abdominal Aortic Aneurysm: Cohort Study of Nearly 500 000 Individuals

**DOI:** 10.1161/CIRCULATIONAHA.120.053022

**Published:** 2021-06-25

**Authors:** Paul Welsh, Claire E. Welsh, Pardeep S. Jhund, Mark Woodward, Rosemary Brown, Jim Lewsey, Carlos A. Celis-Morales, Frederick K. Ho, Daniel F. MacKay, Jason M.R. Gill, Stuart R. Gray, S. Vittal Katikireddi, Jill P. Pell, John Forbes, Naveed Sattar

**Affiliations:** 1 Institute of Cardiovascular and Medical Sciences (P.W., P.S.J., R.B., C.A.C.-M., J.M.R.G., S.R.G., N.S.), University of Glasgow, United Kingdom.; 2 Institute of Health & Wellbeing (J.L., F.K.H., D.F.M., S.V.K., J.P.P.), University of Glasgow, United Kingdom.; 3 Population and Health Sciences Institute, Newcastle University, United Kingdom (C.E.W.).; 4 The George Institute for Global Health, School of Public Health, Imperial College London, United Kingdom (M.W.).; 5 The George Institute for Global Health, University of New South Wales, Sydney, Australia (M.W.).; 6 Department of Epidemiology, Johns Hopkins University, Baltimore, Maryland (M.W.).; 7 University of Limerick, Ireland (J.F.).

**Keywords:** aneurysm, prediction, risk score

## Abstract

**Background:**

Abdominal aortic aneurysm (AAA) can occur in patients who are ineligible for routine ultrasound screening. A simple AAA risk score was derived and compared with current guidelines used for ultrasound screening of AAA.

**Methods:**

United Kingdom Biobank participants without previous AAA were split into a derivation cohort (n=401 820, 54.6% women, mean age 56.4 years, 95.5% White race) and validation cohort (n=83 816). Incident AAA was defined as first hospital inpatient diagnosis of AAA, death from AAA, or an AAA-related surgical procedure. A multivariable Cox model was developed in the derivation cohort into an AAA risk score that did not require blood biomarkers. To illustrate the sensitivity and specificity of the risk score for AAA, a theoretical threshold to refer patients for ultrasound at 0.25% 10-year risk was modeled. Discrimination of the risk score was compared with a model of US Preventive Services Task Force (USPSTF) AAA screening guidelines.

**Results:**

In the derivation cohort, there were 1570 (0.40%) cases of AAA over a median 11.3 years of follow-up. Components of the AAA risk score were age (stratified by smoking status), weight (stratified by smoking status), antihypertensive and cholesterol-lowering medication use, height, diastolic blood pressure, baseline cardiovascular disease, and diabetes. In the validation cohort, over 10 years of follow-up, the C-index for the model of the USPSTF guidelines was 0.705 (95% CI, 0.678–0.733). The C-index of the risk score as a continuous variable was 0.856 (95% CI, 0.837–0.878). In the validation cohort, the USPSTF model yielded sensitivity 63.9% and specificity 71.3%. At the 0.25% 10-year risk threshold, the risk score yielded sensitivity 82.1% and specificity 70.7% while also improving the net reclassification index compared with the USPSTF model +0.176 (95% CI, 0.120–0.232). A combined model, whereby risk scoring was combined with the USPSTF model, also improved prediction compared with USPSTF alone (net reclassification index +0.101 [95% CI, 0.055–0.147]).

**Conclusions:**

In an asymptomatic general population, a risk score based on patient age, height, weight, and medical history may improve identification of asymptomatic patients at risk for clinical events from AAA. Further development and validation of risk scores to detect asymptomatic AAA are needed.

## Abstract 2

Supplemental Digital Content is available in the text.

**Clinical Perspective**

**What Is New?**

- A model based on US Preventive Services Task Force guidelines yielded sensitivity of 63.9% and specificity 71.3% in identifying patients who would potentially benefit from ultrasound on the basis of incidence of abdominal aortic aneurysm (AAA).
- In contrast, a model based on an AAA risk score, with guidance to refer for abdominal ultrasound at a threshold of 0.25% 10-year risk, yielded sensitivity 82.1% and specificity 70.7%.
- A simple 10-year AAA risk score, using routine clinical information without the need for blood tests, therefore gives excellent discrimination of those at risk of adverse outcomes from incident AAA.

**What Are the Clinical Implications?**

- More work needs to be done to develop and test different approaches to refer patients for AAA ultrasound screening. These data suggest that risk score–based approaches are potentially feasible in clinical practice.

An aneurysm is a pathological distension of a section of blood vessel, typically the aorta.^1–4^ Aortic aneurysms can occur anywhere in the aorta’s length, but abdominal aortic aneurysms (AAAs) are associated with increased mortality if rupture occurs (around 50% in those who reach the hospital) because of catastrophic bleeding.^1,5,6^ In the Oxford Vascular Study, among 65- to 74-year-olds, the incidence was 5.5 events per 10 000 person-years in men and 1.1 events per 10 000 person-years in women.^7^ Death from AAA accounts for around 2% of all deaths in men aged 65 and over, and few clinical symptoms are noted in AAA that subsequently rupture.

In the United Kingdom, a routine screening program invites men in the year of their 65th birthday for abdominal ultrasonography, and more recent updates suggest women of age 70 years and over be screened if they have risk factors such as ever smoking, or if they have cardiovascular disease, chronic obstructive pulmonary disease, peripheral arterial disease, hyperlipidemia, hypertension, or a family history of AAA.^8,9^ If an AAA of >5.5 cm diameter is detected, the patient is rapidly referred for surgical intervention, with slower referral for smaller-sized AAA. In 2019, the US Preventive Services Task Force (USPSTF) recommended screening for AAA in men aged 65 to 75 years who have ever smoked. The USPSTF recommends against routine screening in women who have never smoked and have no family history of AAA and states that evidence is insufficient to recommend for or against AAA screening in women with either history of smoking or family history of AAA.^10,11^ Although not all international recommendations agree on a precise screening strategy, others are broadly similar.^12^ Screening for AAA in women has not been demonstrated to be clinically effective.^13^ In considering balancing the risks and benefits of screening, ultrasonography itself has high sensitivity (94%–100%) and specificity (98%–100%) for the detection of AAA, although risk and benefits of surgical intervention for smaller aneurysms must be carefully considered.^8,11^ Although age-based screening thresholds in women may not be cost-effective,^14^ a more refined risk score based on systematic routine clinical data may help improve clinical care.

This study aimed to use a large, well-phenotyped United Kingdom database to develop, and internally validate, an AAA risk score using simple clinical data. It was hypothesized that such a risk score could more precisely discriminate those at risk of adverse outcomes from AAA than current approaches.

## Methods

### Data Source and Cohort Selection

The data used in this study are available via UK Biobank (https://www.ukbiobank.ac.uk/), subject to necessary approvals. UK Biobank is a large population-based cohort study of 502 488 participants ranging in age from 37 to 73 years, recruited between 2006 and 2010.^15,16^ All participants underwent an assessment at 1 of 22 centers across England, Scotland, and Wales, where touch-screen questionnaires recorded health and lifestyle information, and a wide range of biological measurements were taken.

Participants were excluded if they self-reported history of aortic aneurysm, aortic dissection, or cerebral aneurysm (n=447), or if participants had a hospital diagnosis of vascular disease (*International Classification of Diseases, Tenth Revision* [*ICD-10*] codes I71–I73, I77–I79) before or within 30 days after the UK Biobank assessment date (n=2969). After further excluding 13 436 participants with missing data for covariates, there were 485 636 participants with complete data included in the study.

Implementation of the United Kingdom National Health Service (NHS) screening policy for AAA in men aged 65 or over was complete in most parts of the United Kingdom by late 2009.^17^ In this analysis, the data were divided into 2 sets, the derivation and validation sets (ie, a holdout set). The derivation group was those participants who attended their baseline assessment on or before December 31, 2009 (n=401 820). Those whose baseline assessment was on or after January 1, 2010 (n=83 816) were used as a nonrandom holdout internal validation cohort. The nonrandom design of the validation holdout cohort serves 2 purposes. First, it provides an estimate of whether the risk score performs similarly once the new screening approach was implemented. Second, a nonrandom split may be preferable as it reduces the similarity of the 2 sets of participants, thereby strengthening the intended validation.^18^

UK Biobank received ethical approval from the North West Multi-Center Research Ethics Committee (REC reference: 11/NW/03820). All participants gave written informed consent before enrollment, in accordance with the principles of the Declaration of Helsinki. This project was performed under UK Biobank project approval No. 42475.

### Outcome

The NHS in the United Kingdom generates routine data on admissions that includes details of all inpatient admissions at all NHS hospitals, and these hospital records were linked by anonymized numeric participant identification number to UK Biobank participants.^19,20^ All clinical data in the hospital inpatient data were coded according to the World Health Organization’s *ICD-10* codes. All operations and procedures were coded according to the Office of Population, Censuses and Surveys: Classification of Interventions and Procedures codes (OPCS-4). Dates and causes of death were obtained from death certificates held by the NHS Information Center for participants from England and Wales and the NHS Central Register Scotland for participants from Scotland.

The outcome of interest was first hospital inpatient diagnosis of AAA or death from AAA (both based on *ICD-10* codes I71.3 or I71.4), or an AAA-related surgical procedure (Expanded Methods in the Data Supplement). The definition of AAA-related inpatient diagnosis was therefore based on diagnostic codes for AAA but did not relate to specific diagnostic criteria (such as aneurysm size). As a sensitivity analysis to test performance of the model in discriminating the most serious incident AAA cases, a composite outcome was derived for death from AAA (*ICD-10* codes I71.3 or I71.4) or an AAA-related surgical procedure only. In deriving survival models for each participant, the start of the period at risk was the date of assessment, and the period at risk ended at the first qualifying AAA event, or end of follow-up (June 30, 2020, in England; February 29, 2016, in Wales; October 31, 2017, in Scotland), whichever came first. For the 10-year risk score, follow-up was curtailed to a maximum of 10 years for each participant.

### Characteristics Associated With AAA Events

Characteristics considered for a simple AAA risk score (ie, a score that does not involve the use of blood biomarkers, and involves variables routinely collected in clinical data) were age, sex, systolic blood pressure, diastolic blood pressure, pulse pressure, smoking status, alcohol use, height, weight, body mass index, baseline cardiovascular disease (CVD; hospitalization with diagnoses including *ICD-10* codes I20–I25 and I60–I69 occurring before the date of assessment), family history of CVD (self-report of heart disease or stroke in a mother, father, or sibling), baseline diabetes (self-reported type 1 or type 2 diabetes, and those who reported using insulin), chronic kidney disease, atrial fibrillation, rheumatoid arthritis (self-reported), use of blood pressure–lowering medication (self-reported), and cholesterol-lowering medications (self-reported). Rheumatoid arthritis was included in the list of potential characteristics associated with AAA because it is a systemic inflammatory condition that may confer increased risk of AAA.^21^ A further AAA risk score, including the same clinical variables as well as blood-based biomarkers, was developed. Potential biomarkers tested for inclusion were white blood cell count, platelet count, low-density lipoprotein cholesterol, high-density lipoprotein cholesterol, triglycerides, lipoprotein(a) (Lp[a]), liver function tests (aspartate aminotransferase, alanine aminotransferase, alkaline phosphatase, and γ-glutamyl transferase), glucose, cystatin-C, C-reactive protein, and vitamin D. Blood collection sampling procedures for the UK Biobank study have been previously described and validated.^22^ Blood tests were performed at a dedicated central laboratory, using rigorous quality control and external performance monitoring. Further details of these measurements and assay performances can be found in the UK Biobank online showcase and protocol.^22^

Systolic and diastolic blood pressure were measured in each participant, following a standardized protocol. The average of 2 measurements was used, preferentially using an automated reading where available. Pulse pressure was calculated as systolic blood pressure minus diastolic blood pressure. Weight was measured using a Tanita BC418MA body composition analyzer, and body mass index was calculated as weight (kg)/height (m).^2^ Smoking status was categorized as never, former, or current smoker. Postcode of residence was used to determine the Townsend socioeconomic deprivation index at recruitment.^23^ Participants were asked, “What is your ethnic group?” and we defined responses to this as race/ethnicity using categories of White, Black, South Asian, or other.

For comparison with the AAA risk score, models of current clinical practices for screening for AAA with ultrasonography were as follows:

1. A model of current USPSTF guidelines (conduct abdominal ultrasound screening in men and women who have ever smoked, and men who have cardiovascular disease, at age 65–75 years)^11^
2. A model of current UK National Institute for Health and Excellence guidelines (conduct abdominal ultrasound screening in all men at age 66 years, and in women at age 70 years if they have ever smoked, or if they have cardiovascular disease, chronic obstructive pulmonary disease, peripheral arterial disease, hyperlipidemia, or hypertension).^8^
3. A hypothetical model whereby all men are screened at age 65 years, and all women are screened at age 70 years. This model is intended to act a comparative clinical approach where sensitivity is prioritized over specificity for an age-based approach to referral for ultrasound.

Details of the definitions underpinning these models are given in the Expanded Methods in the Data Supplement.

### Statistical Analyses

Continuous variables are presented as mean and SD if approximately symmetrically distributed, and median and interquartile interval (IQI) if skewed. Categorical variables were presented as counts and percentages. Each variable was tested for association with incident AAA, separately in derivation and validation cohorts, using an unpaired *t* test, the Wilcoxon rank-sum test, or a χ^2^ test as appropriate.

A forward model building process was implemented for the “AAA risk score” in the derivation cohort, starting with a model including age, sex, and baseline CVD and comparing models for improvement in Akaike information criterion (>10 U difference) on addition of new variables.^24^ Where there was evidence of potential collinearity (such as with pulse pressure and systolic blood pressure), the variable that fit the model better was preferentially used. Continuous variables were first tested for linearity of the association with AAA using restricted cubic splines, log transforming the marker if required. If the association was not linear, the variable was categorized based on turning or inflection points (this was only the case for diastolic blood pressure, where a binary model split at 90 mm Hg was implemented). Once all variables had been tested and the variables for inclusion finalized, all potential pairwise multiplicative interactions between retained variables were tested for additional inclusion, again on the basis of improving the Akaike information criterion by >10 U. An additional AAA risk score that included clinical variables and blood biomarkers (“AAA risk score with blood biomarkers”) was an extension of the simple AAA risk score, and tested model fit on addition of blood biomarkers, in the same manner. The proportional hazard assumption was tested by visual inspection of Schöenfeld residuals. The final Cox models were then run separately in the validation cohort. The predictive ability of the Cox models, over 10 years of follow-up, was tested by Harrell’s C-index separately in both derivation and validation cohorts, using 2000 bootstraps. These metrics were compared with the C-index from Cox models of current clinical practice (which were time-varying models).

A 10-year AAA risk score was then derived from the Cox models, using the derivation cohort. Predicted 10-year risk was derived for each participant, using appropriate centering for each continuous variable (median age 58 years, weight 76 kg, height 168 cm). The calibration of the risk score was evaluated separately in the validation and derivation cohort, over 10 years, using the stcoxgrp command, as previously described.^25^ A range of binary thresholds (chosen pragmatically based on observed data) were considered as potential “high-risk” thresholds to refer for ultrasound screening (specifically, thresholds at 0.25% 10-year risk, 0.3% 10-year risk, and 0.5% 10-year risk). Using these thresholds, a range of sensitivities, specificities, positive predictive values, and negative predictive values were obtained. The 0.25% threshold was selected as the primary threshold, based on maximizing the sum of observed sensitivity and specificity. The performance of these risk score thresholds was compared with “current clinical practice” models described in Characteristics Associated With AAA Events. We also modeled a “combined approach” whereby all participants in the cohort would be given an AAA risk score at baseline (referring those at 0.30% 10-year risk for ultrasound), and also in parallel referring all participants for ultrasound according to the above model of USPSTF guidelines. Binary net reclassification index (NRI) was also assessed in comparing the performance of specific risk score thresholds to the performance of current clinical practice.

We followed recommendations for Transparent reporting of a multivariable prediction model for individual prognosis or diagnosis (TRIPOD) reporting guidelines for development and validation.^26^ All analyses were performed in STATA (version 15.1) or in R (version 4.0.3) for C-index and NRI analyses.

## Results

### Derivation Cohort

In the derivation cohort of 401 820 participants (54.6% women, mean age 56.4 years, 95.5% White, 1.3% Black, 2.0% South Asian, 1.3% other race) at baseline, 17.5% of women and 20.0% of men were >65 years old, and 0.4% of women and 0.5% of men were >70 years old. Over a median of 11.3 (IQI 10.7, 11.9) years of follow-up, 62.1% of women and 62.8% of men attained an age of at least 65 years, and 43.3% of women and 44.6% of men attained an age of at least 70 years.

**Figure 1.**
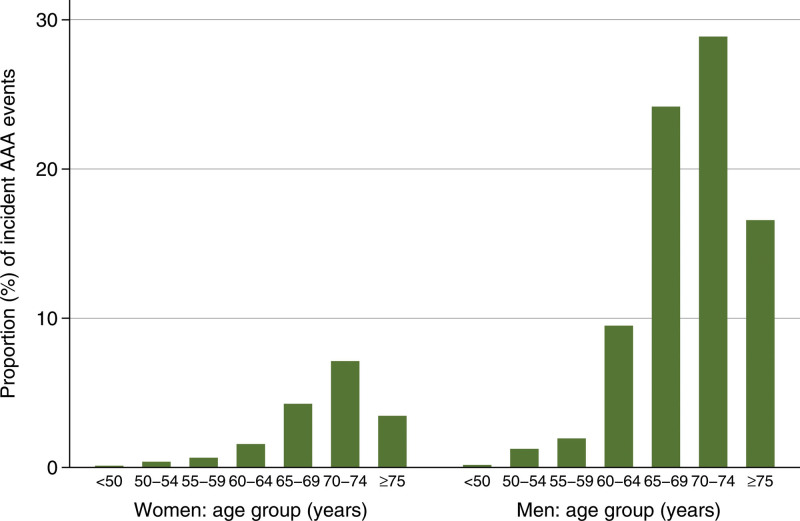
Proportion (%) of incident abdominal aortic aneurysm (AAA) events by sex and by age at which event occurred, in 1291 men and 279 women within the derivation cohort.

There were 1570 (0.4%) incident cases of AAA over the follow-up. Of 279 events in women, 107 (38.4%) occurred before the age of 70 years, and of 1291 events in men, 199 (15.4%) occurred before the age of 65 years (Figure 1). The mean age at which an AAA event occurred was 70.8 years (SD 5.7 years) in women and 70.4 years (SD 5.5 years) in men.

The incidence of AAA was 3.6 (95% CI, 3.4–3.7) per 10 000 person-years, 1.1 (95% CI, 1.0–1.3) per 10 000 person-years in women, and 6.5 (95% CI, 6.1–6.8) in men per 10 000 person-years. In participants aged <65 years at baseline, the incidence was 0.7 (95% CI, 0.6–0.8) per 10 000 person-years in women and 4.0 (95% CI, 3.7–4.3) per 10 000 person-years in men. In participants aged 65 years or older at baseline, the incidence was 3.4 (95% CI, 2.9–4.0) per 10 000 person-years in women and 16.9 (95% CI, 15.7–18.3) in men per 10 000 person-years.

Participants diagnosed with AAA over the follow-up were approximately 7 years older on average at baseline, had a higher proportion of men, had a higher Townsend socioeconomic deprivation index, were taller, and had a more adverse general health profile including a higher proportion of people who smoked, higher blood pressure measurements (despite being more likely to take blood pressure medication), a higher weight and body mass index, a higher proportion of people with diabetes, and a higher proportion of people on cholesterol-lowering medication (Table 1).

**Table 1.**
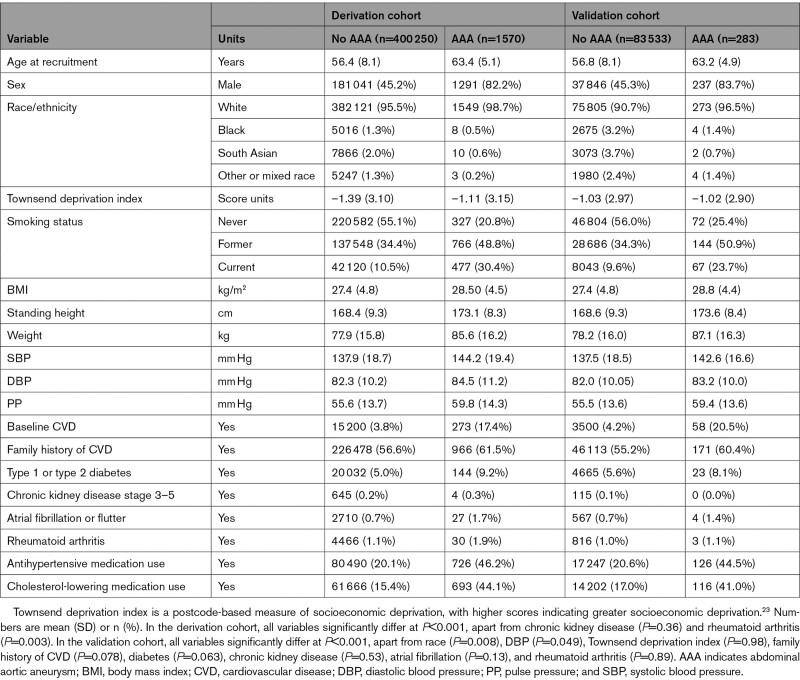
Baseline Characteristics of 401 820 UK Biobank Participants in the Derivation Cohort, and 83 816 Participants in the Validation Cohort, by Incident AAA Status (Using Variables Included in the AAA Risk Score)

### Derivation, Calibration, and Discrimination of the Clinical AAA Risk Score, and Comparison With Current Clinical Practice

Factors associated with AAA included male sex, taller height, a diastolic blood pressure >90 mm Hg, and baseline CVD, whereas baseline diabetes was associated with lower risk of AAA (Table 2). There were also interactions among other risk factors, which were allowed for in the model (Table 2). Specifically, older age was a risk factor, an association that was stronger in current smokers. Heavier weight was a risk factor, an association that was stronger in nonsmokers. Use of blood pressure medication and use of cholesterol-lowering medication were risk factors individually, as well as an interaction between them when taking both (Table 2). The clinical AAA risk score was derived from these risk factors (Expanded Methods in the Data Supplement).

**Table 2.**
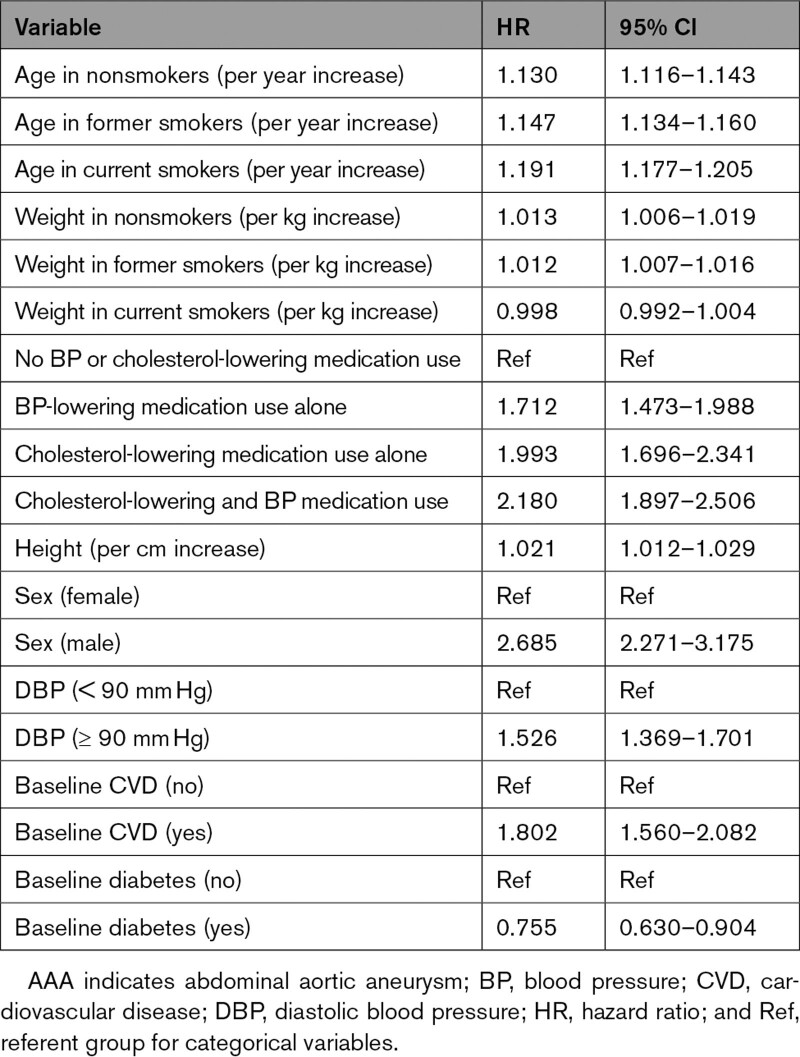
Cox Proportional Hazard Model of Risk Predictors for AAA, Using Variables Included in the AAA Risk Score, in 401 820 Participants in the Derivation Cohort

Over 10 years of follow-up, current clinical practice under the USPSTF guideline model yielded a C-index of 0.738 (95% CI, 0.726–0.751) and had sensitivity of 69.2% and specificity 71.6% in referring participants for abdominal ultrasound before AAA in the derivation cohort (Table 3). The National Institute for Health and Excellence guideline model yielded a C-index of 0.738 (95% CI, 0.725–0.750) and had sensitivity of 72.5% and specificity 63.4% in the derivation cohort (Table 3). The hypothetical strategy to refer all men at age 65 years and all women at age 70 years for ultrasound yielded a C-index of 0.737 (95% CI, 0.726–0.749) and had sensitivity of 78.3% and specificity of 54.8% in the derivation cohort (Table 3).

**Table 3.**
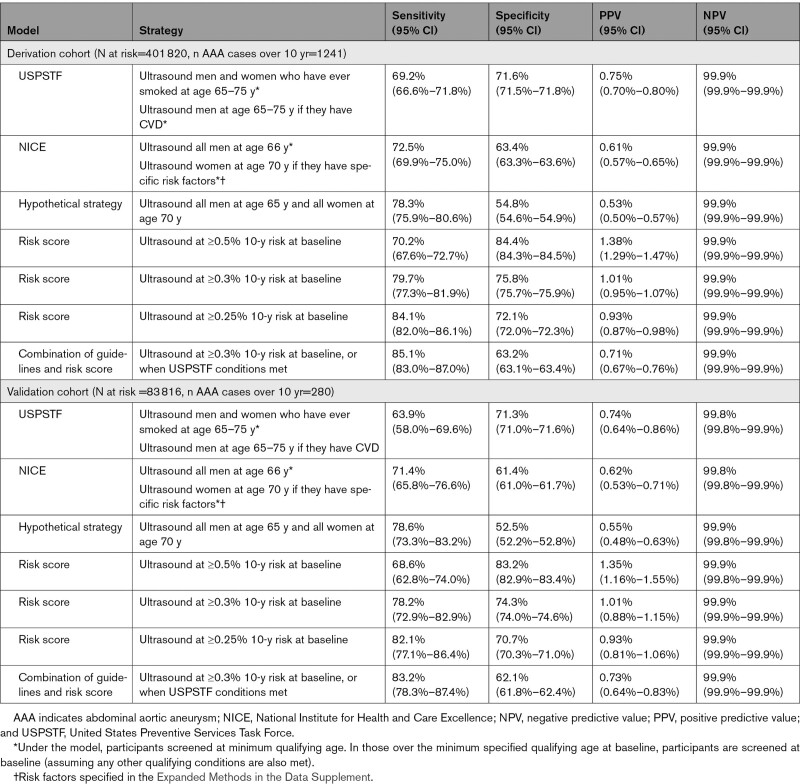
Sensitivity, Specificity, Positive Predictive Value, and Negative Predictive Value of the Current Clinical Practice Models Compared With the AAA Risk Score at Selected Risk Thresholds

**Figure 2.**
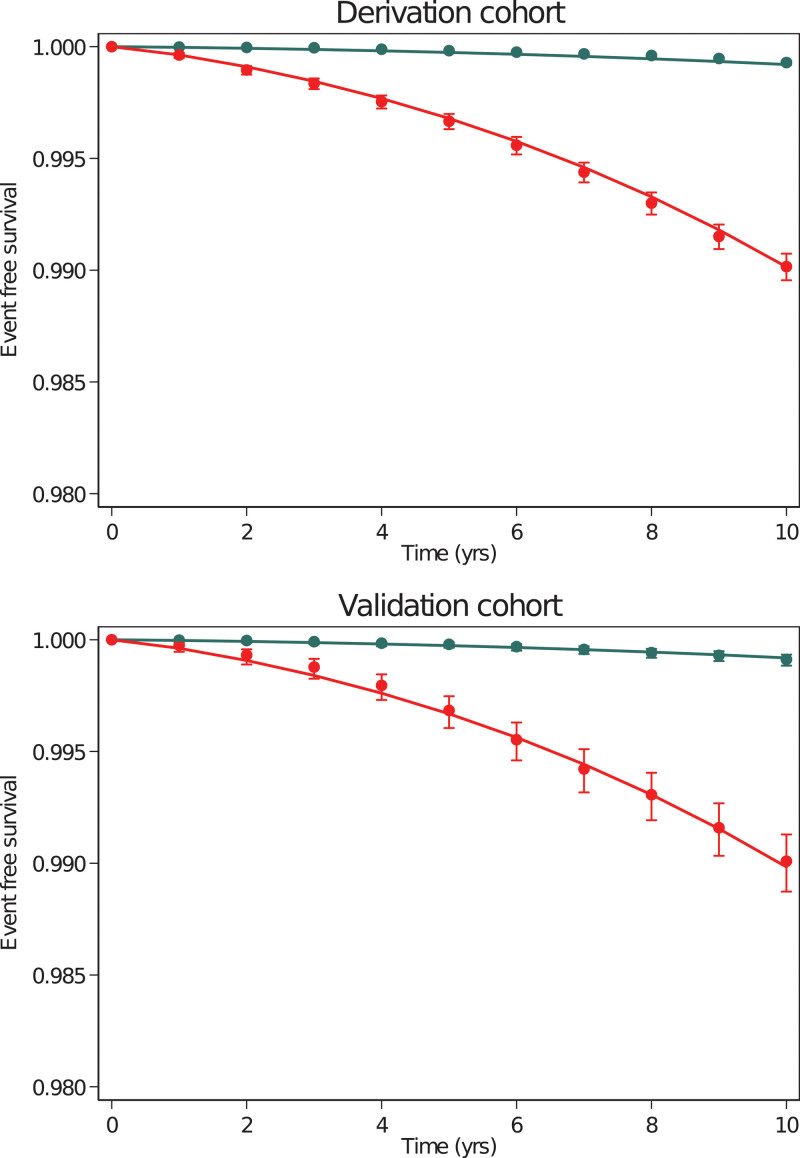
**Abdominal aortic aneurysm risk score calibration in the derivation and validation cohort across 10 years of follow-up.** Curves are predicted survival experience by the risk score, and data points are observed survival with 95% CI. Green curve represents the low-risk group (those at <0.25% 10-year risk: N=289 081 participants, n=197 events in the derivation cohort; N=59 073 participants, n=50 events in the validation cohort) and red curve the high-risk group (those at ≥0.25% 10-year risk: 112 739 participants, n=1044 events in the derivation cohort; N=24 743 participants, n=230 events in the validation cohort).

The median 10-year predicted risk using the AAA risk score was 0.10% (IQI 0.04%, 0.29%) in participants who did not experience AAA during follow-up. The median 10-year predicted risk was 1.13% (IQI 0.40%, 2.37%) in those who experienced AAA during follow-up. The C-index of the AAA risk score was 0.879 (95% CI, 0.870–0.888), and model calibration based on a “high-risk” threshold at 0.25% 10-year risk was good (Figure 2). A binary 0.25% 10-year risk threshold for the AAA risk score had sensitivity 84.1% and specificity 72.1% (Table 3). Choosing a higher risk score threshold (at 0.30% 10-year risk) reduced sensitivity (79.7%) while improving specificity (75.8%), whereas a still higher risk score threshold (at 0.50% 10-year risk) further reduced sensitivity (70.2%) while improving specificity (84.4%; Table 3). A combination approach, encompassing a baseline risk score (referring those at 0.3% 10-year risk) as well as referring participants when they met USPSTF guideline criteria, had sensitivity 85.1%, whereas specificity was 63.2% (Table 3).

The overall categorical NRI was improved under all AAA risk score threshold models compared with the USPSTF guideline model (Table 4). Compared with the USPSTF model, the risk score at any of the 3 thresholds improved the overall NRI. Specifically, the AAA risk score threshold at 0.25% 10-year risk improved both the case NRI +0.149, and the noncase NRI +0.005 (Table 4).

**Table 4.**
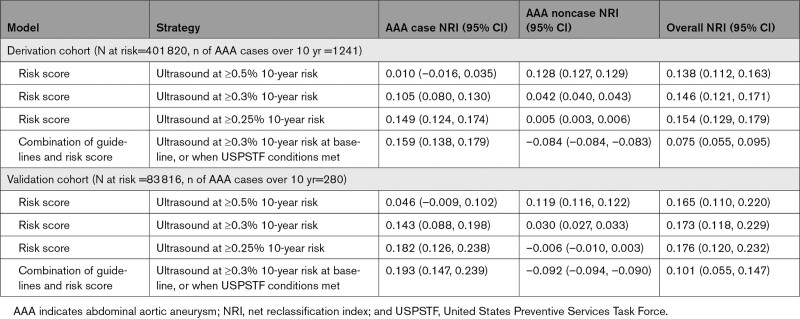
Categorical NRI (Improvement in Decisions to Refer for Ultrasound: Correct Referral for AAA Cases, or Correct Nonreferral for AAA Noncases) Using the AAA Risk Score, at Selected Risk Thresholds, Compared With the USPSTF Model of Current Clinical Practice Only

### Validation of the AAA Risk Score and Comparison With Current Clinical Practice

The validation cohort (83 816 participants, 54.6% women, mean age 56.8 years, 90.8% White, 3.2% Black, 3.7% South Asian, 2.4% other race) had different distributions of demographics and covariates than the derivation cohort. Specifically, the validation cohort was slightly older and more likely to be non-White race; participants were slightly taller, weighed slightly more, and were more likely to have baseline CVD and diabetes than the derivation cohort (Table 1, Table I in the Data Supplement).

In the validation cohort there were 283 (0.34%) cases of AAA over a median of 10.2 years of follow-up (IQI, 10.1–10.3). The incidence of AAA in the validation cohort was 3.4 (95% CI, 3.0–3.8) per 10 000 person-years (similar to the derivation cohort).

Over 10 years of follow-up, current clinical practice under the USPSTF guideline model yielded a C-index of 0.705 (95% CI, 0.678–0.733) and had sensitivity of 63.9% and specificity 71.3% in the validation cohort (Table 3). The National Institute for Health and Excellence guideline model yielded a C-index of 0.719 (95% CI, 0.692–0.745) and had sensitivity of 71.4% and specificity 61.4% in the validation cohort (Table 3). The hypothetical strategy to refer all men at age 65 years and all women at age 70 years for ultrasound yielded a C-index of 0.724 (95% CI, 0.701–0.747) and had sensitivity of 78.6% and specificity 52.5% in the validation cohort (Table 3).

The median 10-year predicted risk was 0.11% (IQI 0.04, 0.31) in participants who did not experience AAA during follow-up in the validation cohort. The median 10-year predicted risk was 0.94% (IQI 0.36, 1.92) in participants who experienced AAA during follow-up. Over 10 years, the C-index of the risk prediction model was 0.856 (95% CI, 0.837–0.878) in the validation cohort; therefore, discrimination from the risk score was slightly lower than in the derivation cohort. Calibration of the risk score was also good in the validation cohort (Figure 2). In the validation cohort, a threshold at 0.25% 10-year risk had sensitivity 82.1% and specificity 70.7%. Higher-risk score thresholds lowered sensitivity but increased specificity (Table 3).

The categorical NRI was improved under the AAA risk score model compared with the USPSTF guideline model in the validation cohort (Table 4). Specifically, the AAA risk score threshold at 0.25% 10-year risk improved both the case NRI +0.182, but not the noncase NRI –0.006 (Table 4).

### Sensitivity Analysis of the AAA Risk Score

The AAA risk score was applied to composite outcome of death from AAA or an AAA-related surgical procedure only, to test discrimination of the most severe incident AAA cases (Table II in the Data Supplement). The AAA risk score thresholds maintained similar sensitivity and specificity to those observed for the primary AAA outcome. The PPV was lower under all strategies (ie, the risk score models and current clinical practice models) because of the lower incidence of cases.

### Derivation and Validation of the AAA Risk Score Including Clinical Variables and Blood Biomarkers

In the AAA risk score including blood biomarkers (N=309 077 participants, n=1237 AAA events in the derivation cohort; N=65 591 participants, n=236 AAA events in the validation cohort; Tables III and IV in the Data Supplement), diabetes was no longer included in the predictive model, but higher C-reactive protein, higher low-density lipoprotein cholesterol, lower high-density lipoprotein cholesterol, higher Lp(a), higher cystatin-C, lower alanine aminotransferase, and lower platelet count were all associated with increased risk of AAA (Table V in the Data Supplement). Model discrimination was good (Figure I in the Data Supplement), with a C-index in the derivation cohort of 0.889 (95% CI, 0.881–0.899) and a C-index in the validation cohort of 0.849 (95% CI, 0.826–0.877). This suggests the AAA risk score including blood biomarkers yielded similar discrimination to the simple AAA risk score in the validation cohort (Tables VI and VII in the Data Supplement).

## Discussion

This study developed and validated a simple AAA risk score, based on data available in routine primary care, for estimating 10-year risk of AAA associated with adverse outcomes in men and women. Using a model in which “high-risk” was defined as a risk of at least 0.25% over 10 years, a risk score–based approach to refer patients for abdominal ultrasound may detect asymptomatic AAA with improved sensitivity and specificity, compared with the existing clinical approaches based on age and sex. This supports the notion that, with availability of simple clinical information plus measures of weight, height, and blood pressure, a simple computer-based algorithm may be able to efficiently recommend referral for abdominal ultrasound using a range of risk factors.

The rates of AAA reported here are in broad agreement with other literature from similar cohorts. For example, in the Oxford Vascular Study, among men and women aged 65 to 74 years, the AAA incidence was 3.2 events per 10 000 person-years.^7^ In UK Biobank, it was 3.6 events per 10 000 person-years. These data are therefore likely identifying similar events to previous work. In addition, the basic risk factors identified in UK Biobank are entirely consistent with existing literature in that AAA is associated with older age, male sex, high blood pressure, smoking, baseline CVD disease, and height.^1,27^ In particular, a separate meta-analysis also reported nonlinear association of diastolic blood pressure with AAA.^28^ Associations of incident AAA with an adverse lipid profile,^1^ poor renal function,^29^ and inflammation^30^ have also been reported. The finding of a continuous association of Lp(a) with AAA is consistent with previous meta-analysis of small studies showing that that Lp(a) may be associated with AAA.^31^ Recent phase 2 trial data show that the drug AKCEA-APO(a)-LRx (also called TQJ230) reduces Lp(a) substantially, with 80% to 90% reductions in patients with established CVD and high Lp(a) levels, and phase 3 trials are underway for CVD prevention.^32^

This study further extends existing data by demonstrating that simple and widely available measures can help guide screening, by abdominal ultrasound, to those that need it most. The information used in this risk score can be easily and inexpensively ascertained by primary care physicians, the patient’s medical history, blood pressure measurement, and other outline primary care data. The option to include routine blood tests in the risk score algorithm may also be of interest, but even a simple risk score based on routine clinical data without the need for blood tests may help guide decision-making. The analysis investigating a dual approach, where risk scoring is conducted as well as age-based referral, suggests improved sensitivity of the referral approach, while only having a moderate impact on specificity. As such, the thresholds reported here present several options to maximize either sensitivity or specificity depending on the specific health care setting.

There are time pressures on primary care physicians, and the burden of risk scoring for various conditions is considerable.^33^ Use of automated software to guide risk scoring and treatment decisions in primary care, with minimal manual input, can be considered where possible. The extra effort required to improve referral practices should be viewed in the context of health care resources saved performing unnecessary ultrasounds.

The strengths of this study include the use of a large, well-phenotyped population, and the use of a composite outcome based on multiple data sources, thereby maximizing the sensitivity of detection. Internal nonrandom validation of the final model was carried out to ensure that it performed well in the cohort assessed after 2009, ie, those where any diagnosis of AAA could have been more likely after screening of eligible men. External validation and development of the risk score reported here are now warranted in other cohorts.

This study has limitations. First, the outcome used to generate the risk score algorithm was a composite of incident AAA diagnosis in hospital, death from AAA, or an AAA-related operation. Given the generally asymptomatic nature of minor AAA, the composite outcome is therefore more likely to represent large or ruptured AAA. Second, no information on aneurysm size was available in the data used in this study; therefore, this information could not be incorporated. Third, although UK Biobank participants are not representative of the general population (and hence cannot be used to provide representative disease prevalence and incidence rates), valid assessment of exposure-disease relationships are nonetheless widely generalizable and do not require participants to be representative of the population at large.^34^ Fourth, UK Biobank has a high proportion of White race participants, and therefore race/ethnicity was not included in the risk prediction model, but may still be an important risk factor. Fifth, the current clinical practice models used here are only approximations of real-life clinical practice. As such, the model is likely to misclassify relative to real-life current screening practices; this would also be true if the risk score is applied in real clinical settings. Sixth, there was no information on which hospital AAA diagnoses occurred because of routine screening in the validation cohort. However, discrimination was similar in the derivation cohort and validation cohort. Seventh, family history of AAA was not available, information on which will further improve AAA prediction in both current clinical practice models and the AAA risk score.^35^ Eighth, there are no current “high-risk” treatment thresholds for AAA screening; the 0.25% 10-year risk threshold (along with other potential “high-risk” thresholds) was based on pragmatic thresholds that illustrate a range of sensitivities and specificities. Other risk thresholds could also be derived from the risk score. Last, the study did not investigate risk factors that have an association with AAA but that are often not routinely coded in primary care, such as physical activity.^36^ The causality of physical activity, and other risk factors, in the prevention of AAA require further study.

### Conclusions

In an asymptomatic general population, a risk score based on patient age, height, weight, and medical history may improve identification of asymptomatic patients at risk for clinical events from AAA. Further development and validation of risk scores to detect asymptomatic AAA are needed.

## Acknowledgments

The authors are grateful to the participants of UK Biobank for allowing use of their data. The authors thank Liz Coyle (University of Glasgow) for her assistance in the preparation of this article.

## Sources of Funding

This work was supported by Chest, Heart, and Stroke Association Scotland (Res16/A165). S.V.K. acknowledges funding from a NHS Research Scotland Senior Clinical Fellowship (SCAF/15/02), the Medical Research Council (MC_UU_00022/2), and the Scottish Government Chief Scientist Office (SPHSU17).

## Disclosures

Dr Welsh reports grant income from Roche Diagnostics, Astrazeneca, Boehringer Ingelheim, and Novartis outside the submitted work. Dr Sattar has received grant and personal fees from Boehringer Ingelheim and personal fees from Amgen, AstraZeneca, Eli Lilly, Merck Sharp & Dohme, Novartis, Novo Nordisk, Pfizer, and Sanofi outside the submitted work. The other authors report no conflicts.

## Supplemental Materials

Expanded Methods

Data Supplement Tables I–VII

Data Supplement Figure I

## Supplementary Material


