# Supplementary material for: Derivation and Validation of a 10-Year Risk Score for Symptomatic Abdominal Aortic Aneurysm: Cohort Study of Nearly 500 000 Individuals
Source: Circulation. 2021 Jun 25;144(8):604–14. doi: 10.1161/CIRCULATIONAHA.120.053022 (PMC8378547; doi:10.1161/CIRCULATIONAHA.120.053022)

SUPPLEMENTAL MATERIAL

Derivation and validation of a 10-year risk score for symptomatic abdominal aortic aneurysm: A cohort study of nearly 500,000 individuals

Welsh et al

Supplemental Expanded Methods

Outcomes

OPCS-4 procedure codes are included in UK Biobank linkage. The following codes are included as an AAA outcome.

L18* - Emergency replacement of aneurysmal segment of aorta

L19* - Other replacement of aneurysmal segment of aorta

L254 - Operations on aneurysm of aorta NEC

L27* - Transluminal insertion of stent graft for aneurysmal segment of aorta

L28* - Transluminal operations on aneurysmal segment of aorta

L464 - Operations on aneurysm of visceral branch of abdominal aorta NEC

Models of current clinical guidelines

For comparison with the AAA risk score, models of current clinical practices for screening for AAA with ultrasonography were defined as:

1) A model approximating current USPSTF guidelines [11] whereby men and women are screened at age 65 (or at baseline if already over the age of 65) if they reported a history of smoking at baseline. This represents a maximally inclusive interpretation of the UPSTF guidelines, since the USPSTF concludes that the evidence is insufficient to determine the net benefit of screening for AAA in women aged 65 to 75 years who have ever smoked. Men who are non-smokers are also screened at age 65 if they experienced CVD (as defined in the main methods section, using hospitalisation data) before the age of 65, or if they experience CVD before baseline if they are already over the age of 65 at baseline. This part of the model is intended to approximate the stipulation that "USPSTF recommends that clinicians selectively offer screening for AAA with ultrasonography in men aged 65 to 75 years who have never smoked".

2) A model approximating current UK National Institute for Health and Excellence (NICE) guidelines [8] whereby men are screened at age 66 (or at baseline if already over the age of 66), regardless of other risk factors. Women are screened at age 70 (or at baseline if already over the age of 70) and if they reported history of smoking, or chronic obstructive pulmonary disease, or peripheral arterial disease, or use of cholesterol lowering medications, or use of blood pressure medications at baseline. Women are also screened at age 70 if they experienced CVD (as defined in the methods section, using hospitalisation data) before the age of 70.

3) A hypothetical model whereby all men are screened at age 65, and all women are screened at age 70. This model is not directly based on existing guidelines and is intended to act a comparative clinical approach where sensitivity is prioritised over specificity for an age-based approach to referral.

In all models of current clinical practice, family history of AAA as a risk factor was not included, as this information is not available in UK Biobank.

AAA Risk score calculation (STATA)

Where:

risk: 10-year AAA risk score (%)

Age_recruitment: Age in years (note below, this is centred on an age of 58 years)

sex: female=0, male =1

smok: smoking status 0=nonsmoker, 1=ex-smoker, 2=current smoker

weight: Weight in Kg (note below, this is centred on a weight of 76kg)

height: Height in cm (note below, this is centred on a height of 168cm)

bpmed: Use of any blood pressure lowering medication, binary, 0=no, 1=yes

statin: Use of any cholesterol lowering medication, binary, 0=no, 1=yes

dbpcat: Diastolic blood pressure ≥90mmHg, binary, 0=no, 1=yes

bcvd: Baseline previous CVD, binary, 0=no, 1=yes

dm_yn: Baseline type 1 or type 2 diabetes, binary, 0=no, 1=yes

and 0. 99942048 is baseline survivor function

```
*In non smokers:
gen nonsmoker = 0.0846679*(Age_recruitment-58) -0.0033033*(weight-76) + 0.5661388*bpmed +
0.8051945*statin -0.500661*bpmed*statin + 1.061764 *sex + 0.0215374*(height-168) +
0.4243123*dbpcat + 0.5927213*bcvdhes - 0.3353556*dm_yn  if smok==0

*In ex smokers:
gen exsmoker = 0.1533288*(Age_recruitment-58) +0.0143366*(weight-76) + 0.5661388*bpmed +
0.8051945*statin -0.500661*bpmed*statin + 1.061764 *sex + 0.0215374*(height-168) +
0.4243123*dbpcat + 0.5927213*bcvdhes - 0.3353556*dm_yn   if smok==1

*In current smokers:
gen smoker = 0.2989571*(Age_recruitment-58) +0.0080073*(weight-76) + 0.5661388*bpmed +
0.8051945*statin -0.500661*bpmed*statin + 1.061764 *sex + 0.0215374*(height-168) +
0.4243123*dbpcat + 0.5927213*bcvdhes - 0.3353556*dm_yn   if smok==2

 gen lin=nonsmoker
 replace lin = exsmoker if lin==.
 replace lin = smoker if lin==.
gen elin = exp(lin)
gen risk = 100*(1-(0. 99942048^elin))
```

Supplemental Tables

Supplemental Table I. Comparison of the derivation and validation cohorts for variables included in the AAA risk score

Factor	Units	Derivation (n=401,820)	Validation (n=83,816)
Age at recruitment	Years	56.4 (8.1)	56.8 (8.1)
Sex	Men	182332 (45.4%)	38083 (45.4%)
Race	White	383670 (95.5%)	76078 (90.8%)
	Black	5024 (1.3%)	2679 (3.2%)
	South Asian	7876 (2.0%)	3075 (3.7%)
	Other	5250 (1.3%)	1984 (2.4%)
Townsend deprivation index	Score units	-1.38 (3.10)	-1.03 (2.97)
Smoking status	Never	220909 (55.0%)	46876 (55.9%)
	Former	138314 (34.4%)	28830 (34.4%)
	Current	42597 (10.6%)	8110 (9.7%)
BMI	kg/m^2	27.4 (4.8)	27.4 (4.8)
Standing height	cm	168.4 (9.3)	168.6 (9.3)
Weight	kg	78.0 (15.8)	78.2 (16.0)
SBP	mmHg	138.0 (18.7)	137.5 (18.5)
DBP	mmHg	82.3 (10.2)	82.0 (10.1)
PP	mmHg	55.6 (13.7)	55.5 (13.6)
Baseline CVD	Yes	15473 (3.9%)	3558 (4.2%)
Family history of CVD	Yes	227444 (56.6%)	46284 (55.2%)
Type 1 or type 2 diabetes	Yes	20176 (5.0%)	4688 (5.6%)
Chronic kidney disease stage 3-5	Yes	649 (0.2%)	115 (0.1%)
Atrial fibrillation or flutter	Yes	2737 (0.7%)	571 (0.7%)
Rheumatoid arthritis	Yes	4496 (1.1%)	819 (1.0%)
Antihypertensive medication use	Yes	81216 (20.2%)	17373 (20.7%)
Cholesterol lowering medication use	Yes	62359 (15.5%)	14318 (17.1%)

AAA abdominal aortic aneurysm; BMI body mass index; cm centimetres; CVD cardiovascular disease; DBP diastolic blood pressure; kg kilograms; m meters; mmHg millimetres of mercury; PP pulse pressure; SBP systolic blood pressure. Townsend deprivation index is a postcode based measure of socioeconomic deprivation, with higher scores indicating greater socioeconomic deprivation. Numbers are mean (sd) or n(%).

Supplemental Table II. Sensitivity analysis of the AAA risk score performance at selected risk thresholds, and compared to current clinical practice models, using the outcome of death from AAA, or AAA related surgical procedures only (i.e., not AAA hospitalisation). This analysis combines both derivation and validation cohorts into a single cohort to maximise power.

Model	Strategy	Sensitivity (95% CI)	Specificity (95% CI)	PPV (95% CI)	NPV (95% CI)
Whole cohort (Number at risk=485,636, number of AAA cases over 10 years=775)					
USPSTF	-Ultrasound men and women who have ever smoked at age 65-75*<br>- Ultrasound men at age 65-75 if they have CVD*	64.4% (60.9, 67.8%)	71.5% (71.4, 71.6%)	0.36% (0.33, 0.39%)	99.9% (99.9, 99.9%)
NICE	- Ultrasound all men at age 66*<br>- Ultrasound women at age 70 if they have specific risk factors*†	68.6% (65.2, 71.9%)	63.0% (62.9, 63.2%)	0.30% (0.27, 0.32%)	99.9% (99.9, 99.9%)
Hypothetical clinical strategy	Ultrasound all men at age 65 and all women at age 70	75.2% (72.0, 78.2%)	54.3% (54.2, 54.5%)	0.26% (0.24, 0.29%)	99.9% (99.9, 99.9%)
Risk score	Ultrasound at ≥0.5% 10-year risk at baseline	69.7% (66.3, 72.9%)	84.1% (84.0, 84.2%)	0.70% (0.64, 0.76%)	99.9% (99.9, 99.9%)
Risk score	Ultrasound at ≥0.3% 10-year risk at baseline	78.5% (75.4, 81.3%)	75.4% (75.3, 75.6%)	0.51% (0.47, 0.55%)	99.9% (99.9, 99.9%)
Risk score	Ultrasound at ≥0.25% 10-year risk at baseline	82.8% (80.0, 85.4%)	71.8% (71.7, 71.9%)	0.47% (0.43, 0.50%)	99.9% (99.9, 99.9%)
Combination of guidelines and risk score	Ultrasound at ≥0.3% 10-year risk at baseline, or when USPSTF conditions met	82.8% (80.0, 85.4%)	63.0% (62.8, 63.1%)	0.36% (0.33, 0.39%)	99.9% (99.9, 99.9%)

CI confidence intervals; NICE National Institute for Health and Care Excellence (UK); NPV negative predictive value; PPP positive predictive value; USPSTF (United States Preventive Services Task Force)

*Under the model, participants screened at minimum qualifying age. In those over the minimum specified qualifying age at baseline, participants are screened at baseline (assuming any other qualifying conditions are also met)

† Risk factors specified in the Expanded Methods in the Supplement

Supplemental Table III. Baseline characteristics of 309,077 UK Biobank participants in the derivation cohort by incident AAA status (for AAA risk score with blood biomarkers).

**Factor**	**Units**	**No AAA (n=307,840)**	**AAA (n=1237)**
Age at recruitment	Years	56.3 (8.1)	63.4 (5.1)
Sex	Men	143631 (46.7%)	1028 (83.1%)
Race	White	294089 (95.5%)	1221 (98.7%)
	Black	3782 (1.2%)	6 (0.5%)
	South Asian	6009 (2.0%)	9 (0.7%)
	Other	3960 (1.3%)	1 (0.1%)
Townsend deprivation index at recruitment		-1.40 (3.09)	-1.16 (3.14)
Smoking status	Never	169459 (55.0%)	256 (20.7%)
	Former	106019 (34.4%)	607 (49.1%)
	Current	32362 (10.5%)	374 (30.2%)
BMI	kg/m^2	27.4 (4.8)	28.5 (4.5)
Standing height	cm	168.7 (9.3)	173.3 (8.3)
Weight	kg	78.1 (15.9)	85.8 (16.1)
SBP	mmHg	137.8 (18.6)	144.5 (19.2)
DBP	mmHg	82.3 (10.1)	84.7 (11.2)
PP	mmHg	55.5 (13.6)	59.8 (14.3)
Baseline CVD	Yes	11935 (3.9%)	217 (17.5%)
Family history of CVD	Yes	173905 (56.5%)	763 (61.7%)
Type 1 or type 2 diabetes	Yes	15790 (5.1%)	111 (9.0%)
Chronic kidney disease stage 3-5	Yes	488 (0.2%)	2 (0.2%)
Atrial fibrillation or flutter	Yes	2141 (0.7%)	23 (1.9%)
Rheumatoid arthritis	Yes	3437 (1.1%)	22 (1.8%)
Antihypertensive medication use	Yes	63802 (20.7%)	599 (48.4%)
Cholesterol lowering medication use	Yes	49231 (16.0%)	546 (44.1%)
**Blood tests**			
White blood cell count	10^9 cells/L	6.60 (5.60, 7.80)	7.40 (6.24, 8.63)
Platelet count	10^9 cells/L	250.70 (216.00, 289.90)	232.40 (199.80, 272.00)
LDL-cholesterol	mmol/L	3.49 (0.78)	3.31 (0.87)
HDL-cholesterol	mmol/L	1.44 (0.37)	1.21 (0.31)
Triglycerides	mmol/L	1.47 (1.04, 2.13)	1.79 (1.28, 2.56)
Lipoprotein(a)	nmol/L	19.10 (7.47, 72.75)	26.80 (9.14, 112.10)
AST	U/L	24.30 (20.90, 28.70)	24.90 (21.30, 29.20)
ALT	U/L	20.00 (15.30, 27.25)	20.60 (16.03, 27.67)
ALP	U/L	79.90 (66.80, 95.20)	84.70 (70.40, 101.40)
GGT	U/L	26.30 (18.50, 40.90)	33.40 (23.60, 49.20)
Glucose	mmol/L	4.91 (4.57, 5.30)	4.95 (4.60, 5.41)
Cystatin-C	mg/L	0.89 (0.80, 0.98)	1.00 (0.91, 1.12)
C-reactive protein	mg/L	1.32 (0.65, 2.75)	2.20 (1.08, 4.37)
Vitamin D	nmol/L	47.60 (32.90, 63.10)	47.10 (32.60, 62.90)

AAA abdominal aortic aneurysm; ALP Alkaline phosphatase; ALT Alanine aminotransferase; AST Aspartate aminotransferase; BMI body mass index; cm centimetres; CVD cardiovascular disease; DBP diastolic blood pressure; GGT Gamma glutamyltransferase; HDL high density lipoprotein; kg kilograms; LDL low density lipoprotein; m meters; mmHg millimetres of mercury; PP pulse pressure; SBP systolic blood pressure.

Values are mean (standard deviation), median (25th, 75th percentile), or number (%).

Supplemental Table IV. Baseline characteristics of 65,591 UK Biobank participants in the validation cohort by incident AAA status (for AAA risk score with blood biomarkers).

**Factor**	**Units**	**No AAA (n=65,355)**	**AAA (n=236)**
Age at recruitment	Years	56.7 (8.2)	63.0 (5.2)
Sex	Men	30445 (46.6%)	199 (84.3%)
Race	White	59335 (90.8%)	227 (96.2%)
	Black	2012 (3.1%)	3 (1.3%)
	South Asian	2423 (3.7%)	2 (0.8%)
	Other	1585 (2.4%)	4 (1.7%)
Townsend deprivation index at recruitment		-1.02 (2.97)	-1.12 (2.80)
Smoking status	Never	36470 (55.8%)	65 (27.5%)
	Former	22579 (34.5%)	121 (51.3%)
	Current	6306 (9.6%)	50 (21.2%)
BMI	kg/m^2	27.4 (4.8)	28.9 (4.5)
Standing height	cm	168.8 (9.3)	174.0 (8.4)
Weight	kg	78.4 (16.0)	87.7 (16.3)
SBP	mmHg	137.2 (18.4)	142.3 (16.8)
DBP	mmHg	81.8 (10.0)	83.3 (10.2)
PP	mmHg	55.4 (13.5)	59.0 (13.9)
Baseline CVD	Yes	2806 (4.3%)	51 (21.6%)
Family history of CVD	Yes	36045 (55.2%)	148 (62.7%)
Type 1 or type 2 diabetes	Yes	3760 (5.8%)	19 (8.1%)
Chronic kidney disease stage 3-5	Yes	93 (0.1%)	0 (0.0%)
Atrial fibrillation or flutter	Yes	462 (0.7%)	3 (1.3%)
Rheumatoid arthritis	Yes	635 (1.0%)	2 (0.8%)
Antihypertensive medication use	Yes	13763 (21.1%)	105 (44.5%)
Cholesterol lowering medication use	Yes	11602 (17.8%)	102 (43.2%)
**Blood tests**			
White blood cell count	10^9 cells/L	6.80 (5.76, 8.00)	7.61 (6.15, 9.23)
Platelet count	10^9 cells/L	234.10 (201.60, 270.20)	216.95 (186.70, 259.85)
LDL-cholesterol	mmol/L	3.46 (0.78)	3.29 (0.84)
HDL-cholesterol	mmol/L	1.47 (0.38)	1.26 (0.35)
Triglycerides	mmol/L	1.42 (1.02, 2.03)	1.80 (1.18, 2.49)
Lipoprotein(a)	nmol/L	19.90 (7.70, 74.00)	27.19 (8.44, 111.90)
AST	U/L	24.60 (21.20, 29.10)	25.10 (21.45, 30.70)
ALT	U/L	20.30 (15.58, 27.44)	21.78 (15.59, 28.71)
ALP	U/L	80.60 (67.50, 96.00)	84.50 (70.90, 106.90)
GGT	U/L	25.80 (18.30, 39.60)	33.90 (23.30, 49.45)
Glucose	mmol/L	5.01 (4.71, 5.35)	5.04 (4.77, 5.43)
Cystatin-C	mg/L	0.88 (0.80, 0.98)	1.03 (0.90, 1.13)
C-reactive protein	mg/L	1.27 (0.63, 2.65)	2.09 (0.95, 4.88)
Vitamin D	nmol/L	42.80 (29.30, 58.50)	44.20 (31.80, 58.30)

AAA abdominal aortic aneurysm; ALP, Alkaline phosphatase; ALT, Alanine aminotransferase; AST, Aspartate aminotransferase; BMI body mass index; CVD cardiovascular disease; DBP diastolic blood pressure; GGT; Gamma glutamyltransferase, HDL, high density lipoprotein; LDL, low density lipoprotein; PP pulse pressure; SBP systolic blood pressure.
Values are mean (standard deviation), median (25th, 75th percentile), or number (%).

Supplemental Table V. Cox proportional hazard model of risk predictors, in the AAA risk score including blood biomarkers, in 309,077 participants in the derivation cohort

Variable	HR	95% CI
Age in non-smokers (per year increase)	1.115	1.099-1.130
Age in former smokers (per year increase)	1.131	1.117-1.146
Age in current smokers (per year increase)	1.172	1.156-1.188
Weight in non-smokers (per Kg increase)	1.000	0.993-1.008
Weight in former smokers (per Kg increase)	0.999	0.994-1.005
Weight in current smokers (per Kg increase)	0.985	0.978-0.992
No BP or cholesterol lowering medication use	Ref	Ref
BP lowering medication use alone	1.720	1.455-2.035
Cholesterol lowering medication use alone	2.125	1.738-2.599
Cholesterol lowering and BP medication use	2.440	2.043-2.915
Height (per cm increase)	1.034	1.024-1.044
Sex (female)	Ref	Ref
Sex (male)	2.199	1.803-2.682
DBP (< 90mmHg)	Ref	Ref
DBP (≥ 90mmHg)	1.621	1.433-1.834
Baseline CVD (no)	Ref	Ref
Baseline CVD (yes)	1.624	1.379-1.913
Log CRP (per 1 ln (mg/L) increase)	1.274	1.204-1.349
LDL-cholesterol (per 1 mmol/L increase)	1.223	1.125-1.330
HDL-cholesterol (per 1 mmol/L increase)	0.370	0.297-0.461
Lp(a) (per 50nmol/L increase)	1.123	1.085-1.162
Log Cystatin C (per 1 ln (mg/L) increase)	2.501	1.835-3.408
Log ALT (per 1 ln (U/L) increase)	0.646	0.560-0.745
Platelet count (per 100*10^9 cells/L increase)	0.807	0.730-0.893

CI confidence intervals; cm centimetres; CVD cardiovascular disease; DBP diastolic blood pressure; kg kilograms; ln natural logarithm; m meters; mmHg millimetres of mercury

Supplemental Table VI. Sensitivity, specificity, positive predictive value, and negative predictive value of the current clinical practice models compared to the AAA risk score with blood biomarkers at selected risk thresholds.

Model	Strategy	Sensitivity	Specificity	PPV	NPV
Derivation cohort (Number at risk=309,077, number of AAA cases=974)					
USPSTF	-Ultrasound men and women who have ever smoked at age 65-75*<br>- Ultrasound men at age 65-75 if they have CVD*	69.3% (66.3, 72.2%)	71.6% (71.4, 71.7%)	0.77% (0.71, 0.83%)	99.9% (99.8, 99.9%)
NICE	- Ultrasound all men at age 66*<br>- Ultrasound women at age 70 if they have specific risk factors*†	73.2% (70.3, 76.0%)	63.0% (62.8, 63.1%)	0.62% (0.58, 0.67%)	99.9% (99.8, 99.9%)
Hypothetical clinical strategy	Ultrasound men at age 65 and women at age 70	78.0% (75.3, 80.6%)	54.7% (54.5, 54.9%)	0.54% (0.50, 0.58%)	99.9% (99.9, 99.9%)
Risk score	Ultrasound at ≥0.5% 10-year risk at baseline	73.6% (70.7, 76.4%)	85.2% (85.0, 85.3%)	1.54% (1.43, 1.66%)	99.9% (99.9, 99.9%)
Risk score	Ultrasound at ≥0.3% 10-year risk at baseline	83.1% (80.6, 85.4%)	77.1% (77.0, 77.3%)	1.14% (1.06, 1.22%)	99.9% (99.9, 99.9%)
Risk score	Ultrasound at ≥0.25% 10-year risk at baseline	86.0% (83.7, 88.2%)	73.8% (73.6, 73.9%)	1.03% (0.96, 1.10%)	99.9% (99.9, 99.9%)
Combination of guidelines and risk score	Ultrasound at ≥0.3% 10-year risk at baseline, or when USPSTF conditions met	88.0% (85.8, 90.0%)	63.6% (63.5, 63.8%)	0.76% (0.71, 0.81%)	99.9% (99.9, 99.9%)
Validation cohort (Number at risk=65,591, number of AAA cases=233)					
USPSTF	-Ultrasound men and women who have ever smoked at age 65-75*<br>- Ultrasound men at age 65-75 if they have CVD*	60.5% (53.9, 66.8%)	71.2% (70.8, 71.5%)	0.74% (0.63, 0.88%)	99.8% (99.8, 99.8%)
NICE	- Ultrasound all men at age 66*<br>- Ultrasound women at age 70 if they have specific risk factors*†	69.5% (63.2, 75.4%)	60.8% (60.4, 61.2%)	0.63% (0.54, 0.73%)	99.8% (99.8, 99.9%)
Hypothetical clinical strategy	Ultrasound men at age 65 and women at age 70	76.8% (70.9, 82.1%)	52.3% (52.0, 52.7%)	0.57% (0.49, 0.66%)	99.8% (99.8, 99.9%)
Risk score	Ultrasound at ≥0.5% 10-year risk at baseline	66.1% (59.6, 72.1%)	84.3% (84.0, 84.6%)	1.48% (1.26, 1.73%)	99.9% (99.8, 99.9%)
Risk score	Ultrasound at ≥0.3% 10-year risk at baseline	74.7% (68.6, 80.1%)	76.3% (76.0, 76.6%)	1.11% (0.95, 1.29%)	99.9% (99.8, 99.9%)
Risk score	Ultrasound at ≥0.25% 10-year risk at baseline	76.8% (70.9, 82.1%)	72.9% (72.5, 73.2%)	1.00% (0.86, 1.16%)	99.9% (99.9, 99.9%)
Combination of guidelines and risk score	Ultrasound at ≥0.3% 10-year risk at baseline, or when USPSTF conditions met	81.1% (75.5%, 85.9%)	62.8% (62.5%, 63.2%)	0.77% (0.67, 0.89%)	99.9% (99.9, 99.9%)

*Under the model, participants screened at minimum qualifying age. In those over the minimum specified qualifying age at baseline, participants are screened at baseline (assuming any other qualifying conditions are also met)

† Risk factors specified in Expanded Methods in the Supplement

Supplemental Table VII. Categorical NRI (improvement in decisions to refer for ultrasound: correct referral for AAA cases, or correct non-referral for AAA non-cases) for the AAA risk score including blood biomarkers, at selected risk thresholds, compared to the USPSTF model of current clinical practice.

**Model**	**Strategy**	**AAA case NRI (95% CI)**	**AAA non-case NRI (95% CI)**	**Overall NRI (95% CI)**
Derivation cohort (Number at risk=309,077, number of AAA cases over 10 years=974)				
Risk score	Ultrasound at ≥0.5% 10-year risk	0.043 (0.014, 0.072)	0.136 (0.134, 0.137)	0.179 (0.150, 0.208)
Risk score	Ultrasound at ≥0.3% 10-year risk	0.138 (0.108, 0.167)	0.055 (0.054, 0.057)	0.193 (0.164, 0.222)
Risk score	Ultrasound at ≥0.25% 10-year risk	0.167 (0.139, 0.196)	0.022 (0.020, 0.024)	0.189 (0.161, 0.218)
Combination of guidelines and risk score	Ultrasound at ≥0.3% 10-year risk at baseline, or when USPSTF conditions met	0.187 (0.162, 0.211)	-0.080 (-0.080, -0.079)	0.107 (0.083, 0.132)
Validation cohort (Number at risk=65,591, number of AAA cases over 10 years =233)				
Risk score	Ultrasound at ≥0.5% 10-year risk	0.056 (-0.008, 0.120)	0.132 (0.128, 0.135)	0.187 (0.123, 0.252)
Risk score	Ultrasound at ≥0.3% 10-year risk	0.142 (0.077, 0.206)	0.051 (0.048, 0.055)	0.193 (0.128, 0.257)
Risk score	Ultrasound at ≥0.25% 10-year risk	0.163 0.098, 0.228)	0.017 (0.013, 0.021)	0.180 (0.115, 0.245)
Combination of guidelines and risk score	Ultrasound at ≥0.3% 10-year risk at baseline, or when USPSTF conditions met	0.206 (0.154, 0.258)	-0.083 (-0.086, -0.081)	0.123 (0.071, 0.175)

AAA abdominal aortic aneurysm; CI confidence intervals; NRI net reclassification index; USPSTF (United States Preventive Services Task Force)

Supplemental Figures

Supplemental Figure I AAA risk score with blood biomarkers: calibration in the derivation and validation cohort across ten years of follow up. Curves are predicted survival experience by the risk score, and data points are observed survival with 95% CI. Green curve represents the low risk group (those at <0.25% 10-year risk: N=227,460 participants, n=136 AAA events in the derivation cohort; N=47,677 participants, n=54 AAA events in the validation cohort) and red curve represents the high risk group (those at ≥0.25% 10-year risk: 81,617 participants, n=838 AAA events in the derivation cohort; N=17,914 participants, n=179 AAA events in the validation cohort).

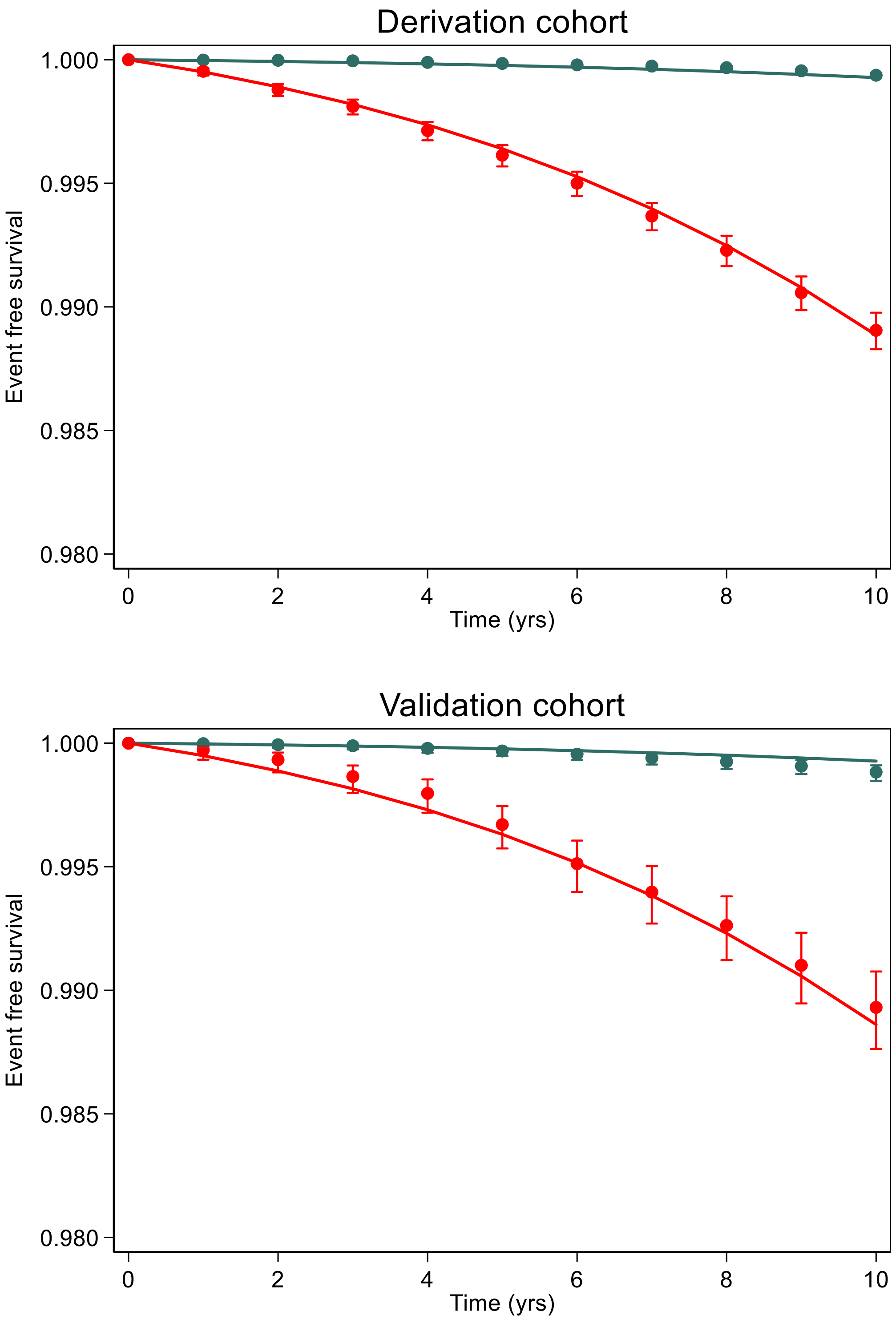

Supplement: Supplementary file 1 [file cir-144-604-s001.pdf]
